# Supplementary figures and images for: Syk activation during FcγR-mediated phagocytosis involves Syk palmitoylation and desulfenylation
Source: Life Sci Alliance. 2026 Feb 4;9(4):e202503500. doi: 10.26508/lsa.202503500 (PMC12872395; doi:10.26508/lsa.202503500)

Figure 1

Panel A

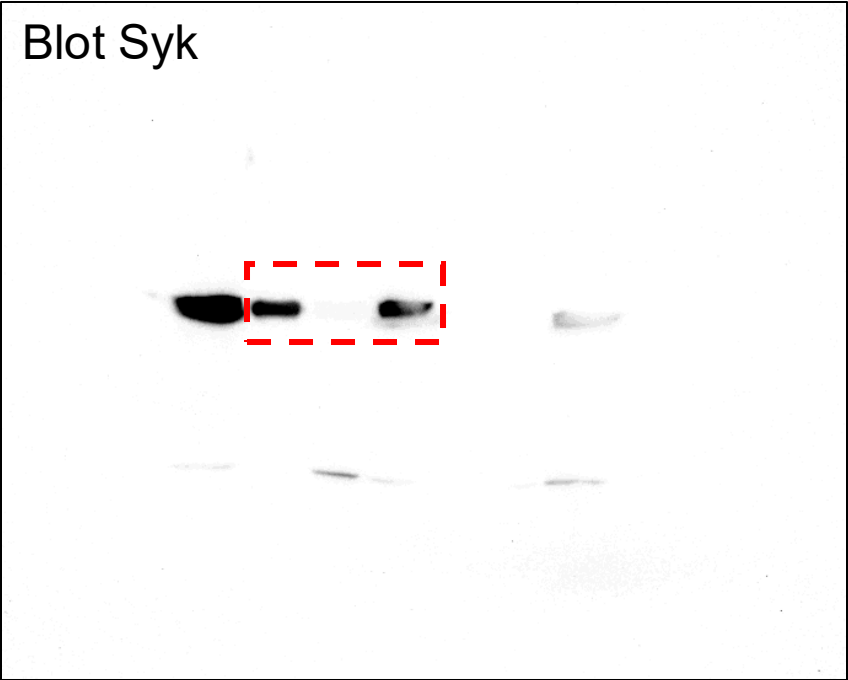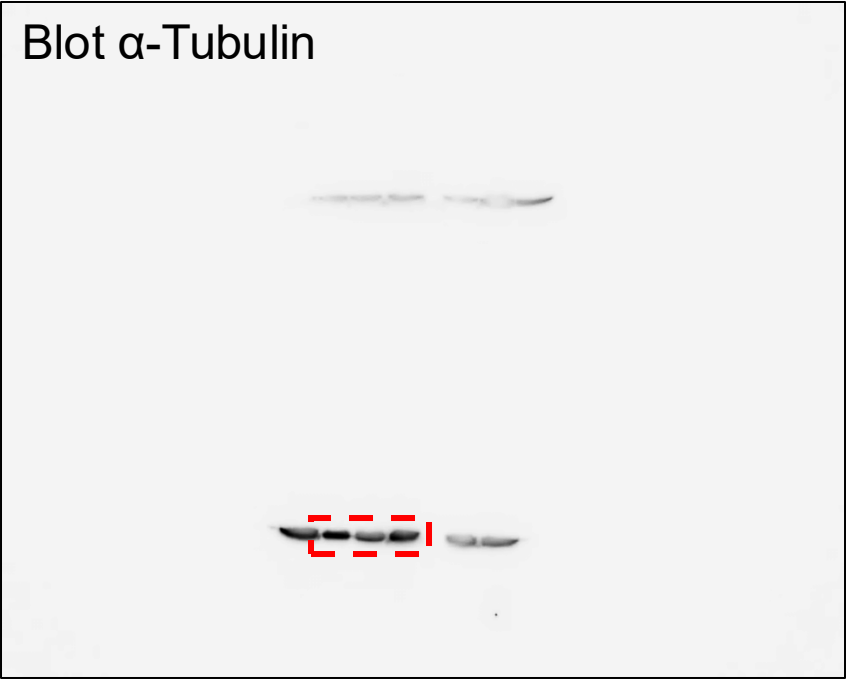

Supplement: Supplementary file 3 [file LSA-2025-03500_SdataF1.3.pdf]

Figure 2

Panel A

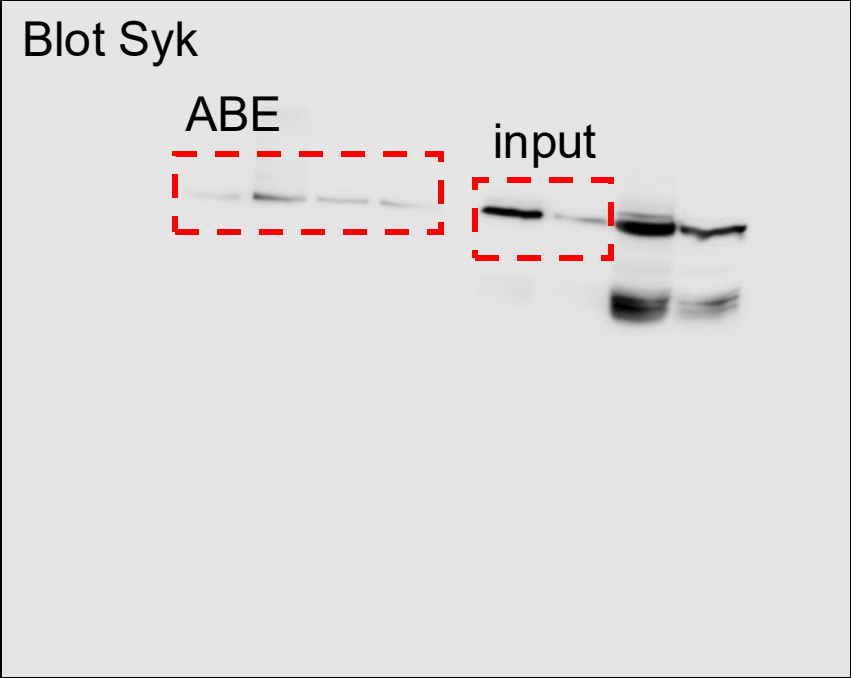

Figure 2

Panel B

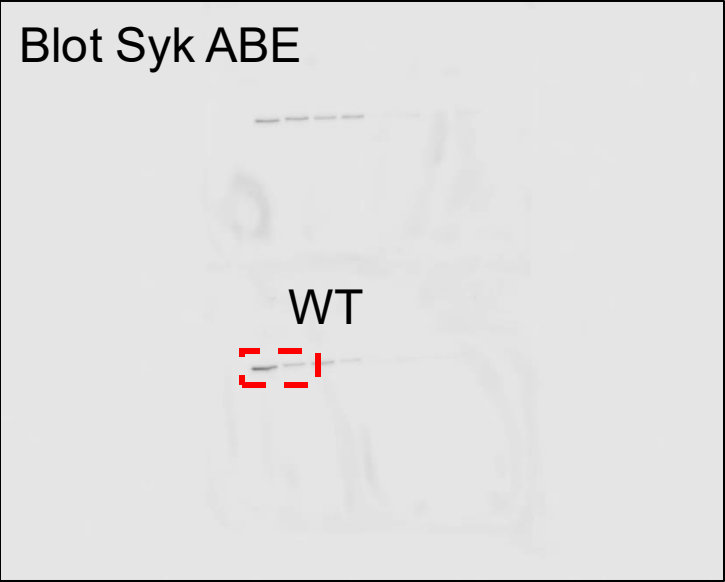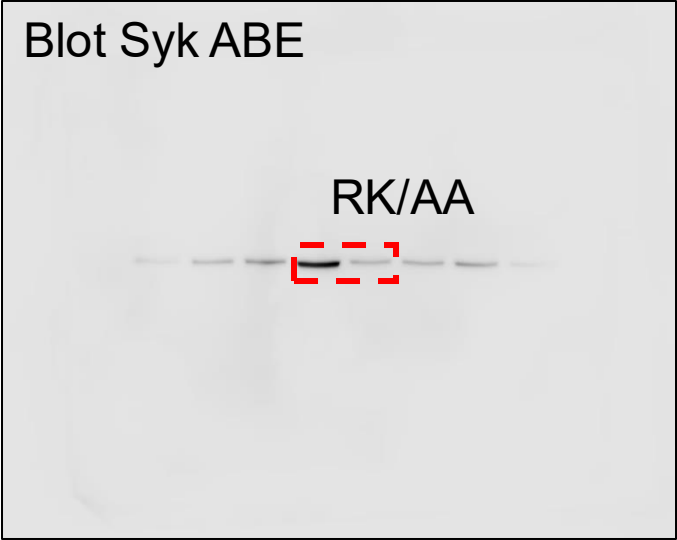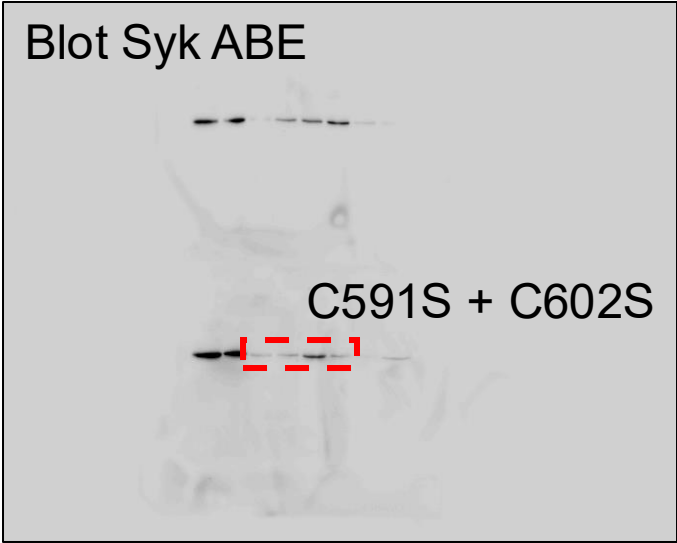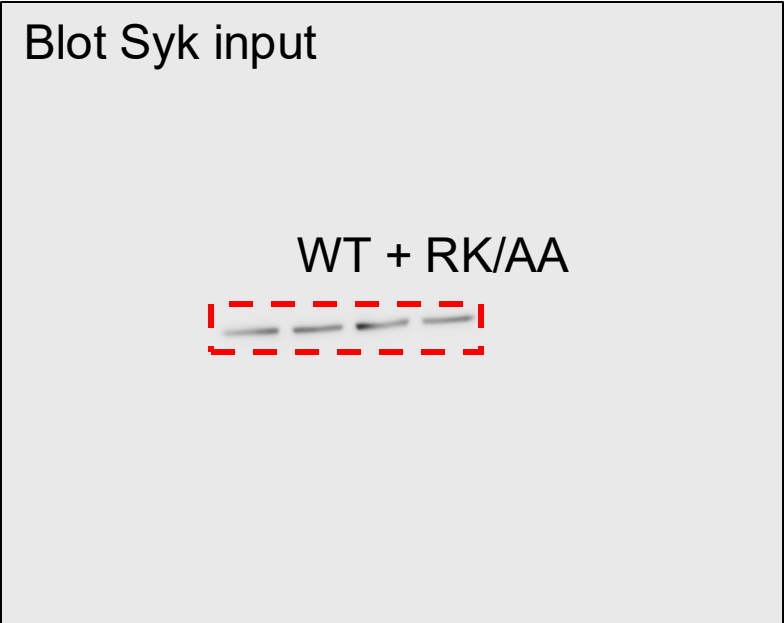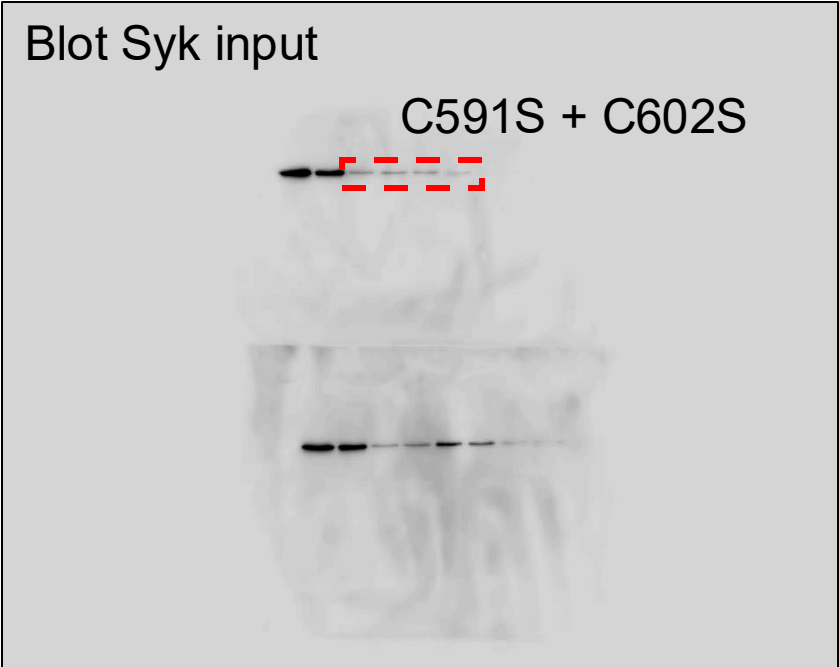

Supplement: Supplementary file 6 [file LSA-2025-03500_SdataF2.4.pdf]

**Figure 3**

Panel A

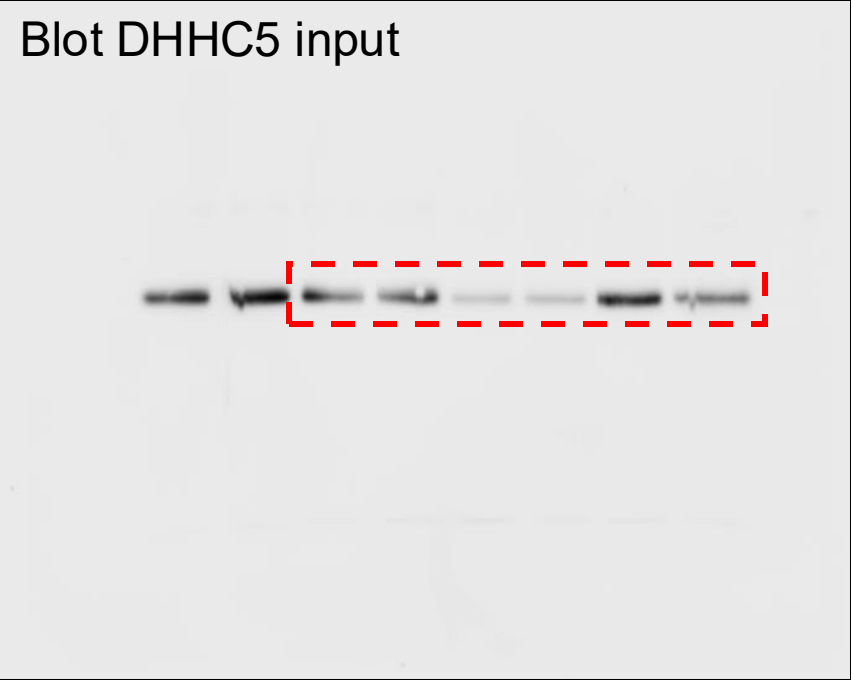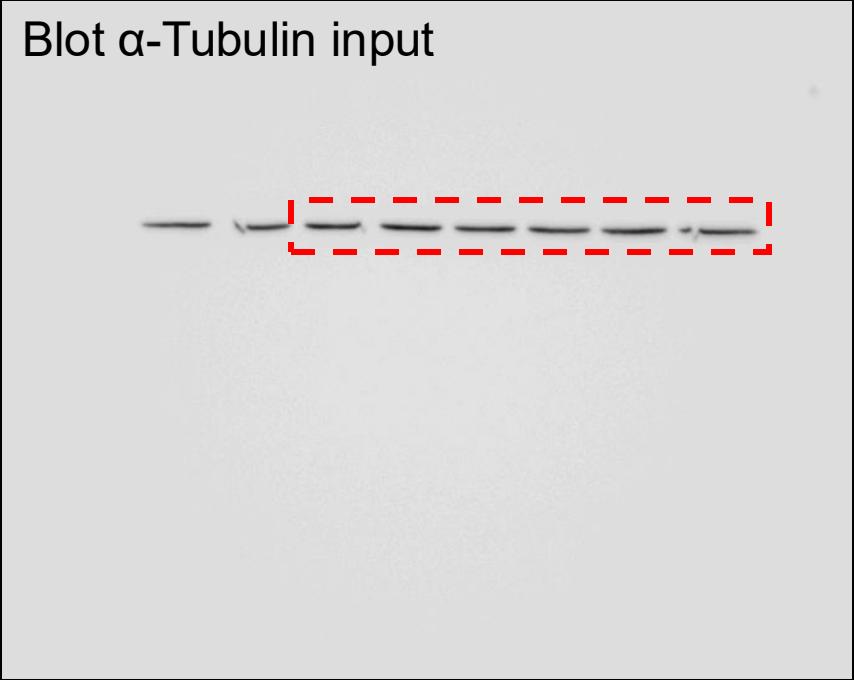

Figure 3

Panel C

Blot Syk ABE

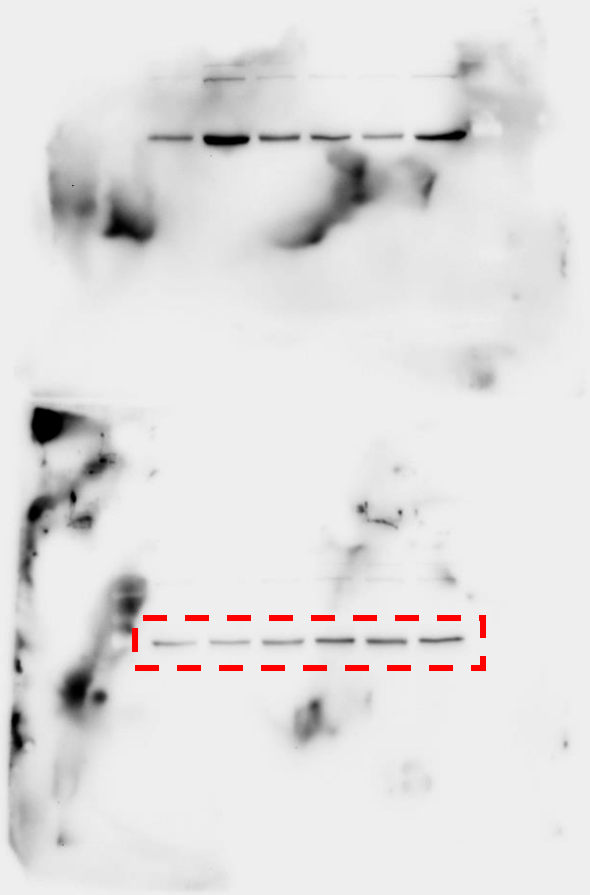

Blot Syk input

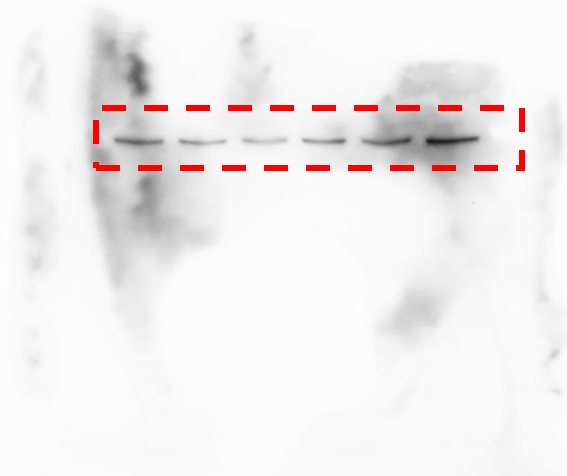

**Figure 3**

Panel E

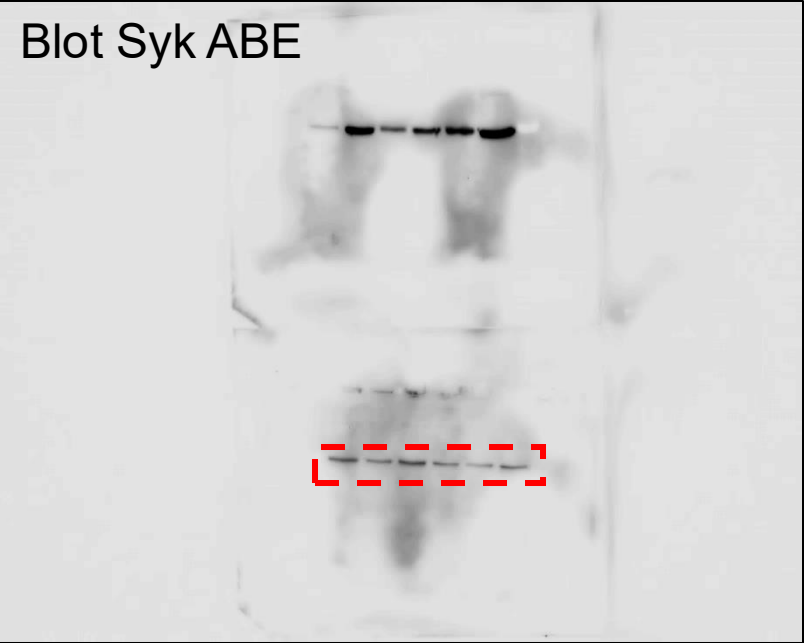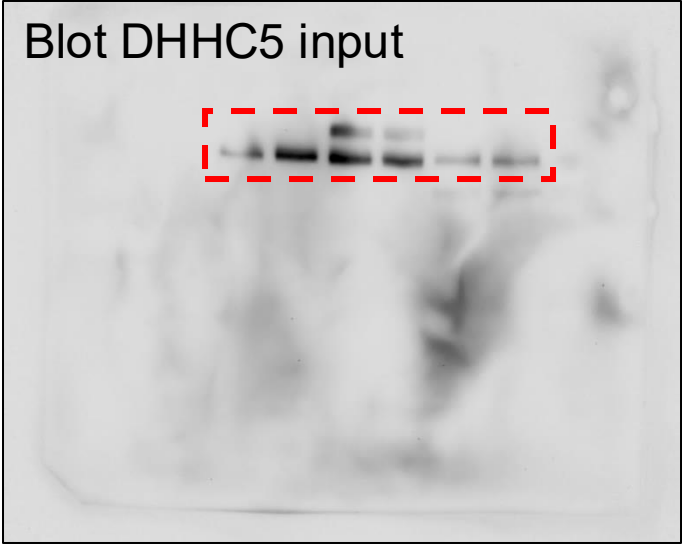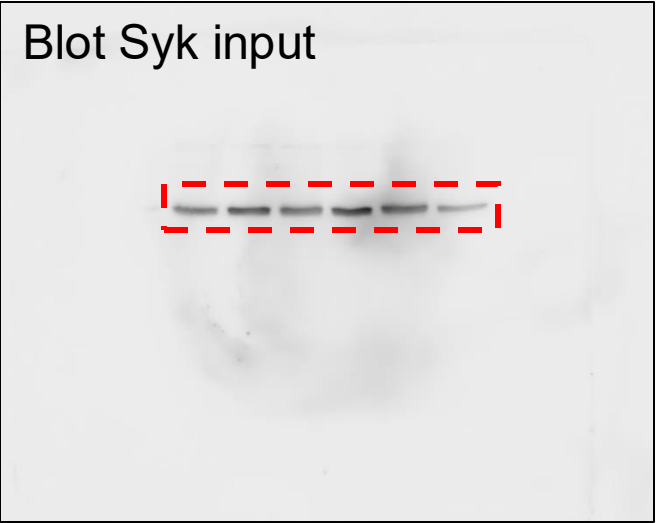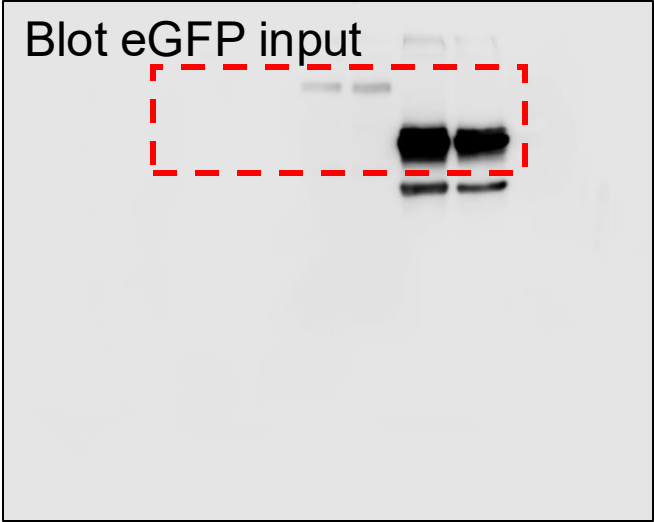

Supplement: Supplementary file 7 [file LSA-2025-03500_SdataF3.4.pdf]

Figure 4

Panel A

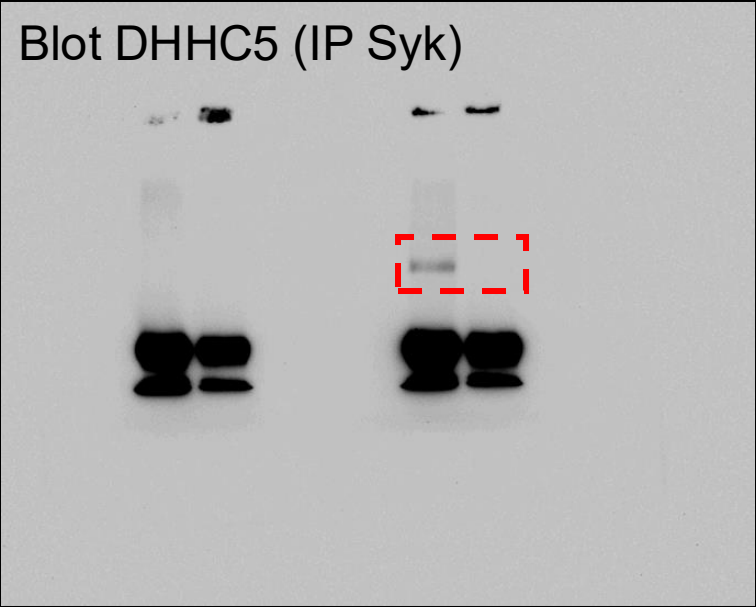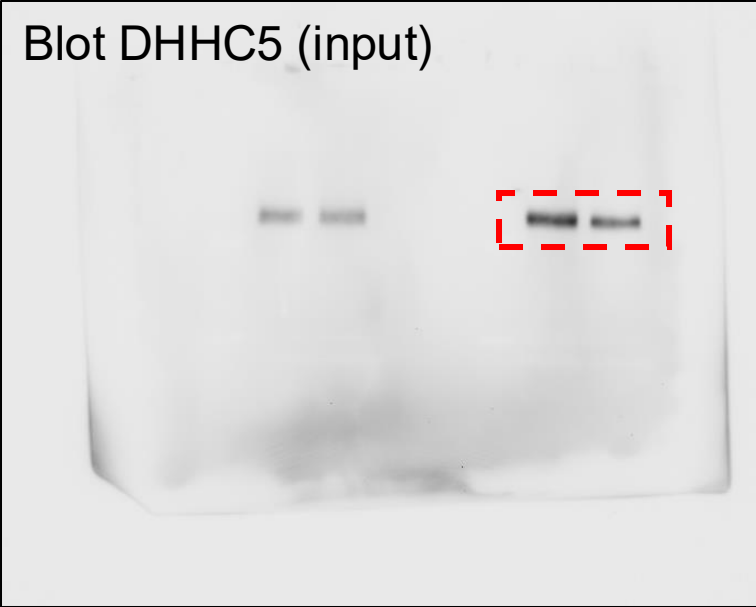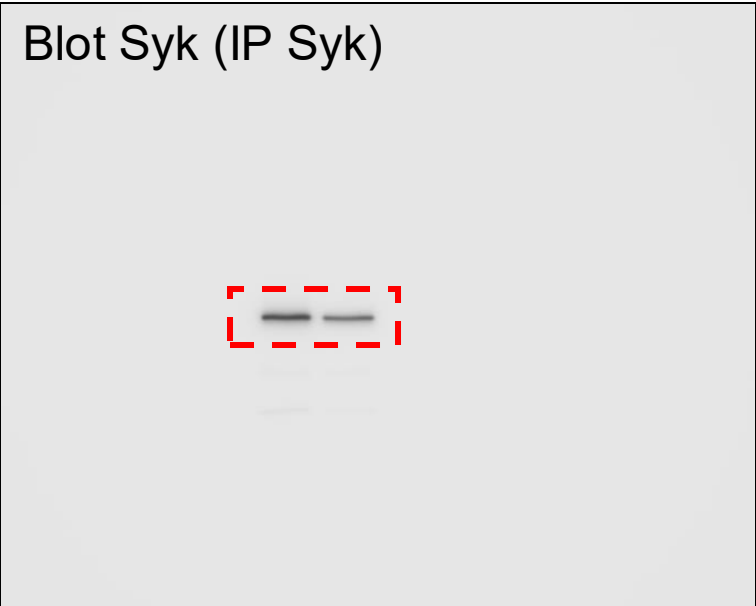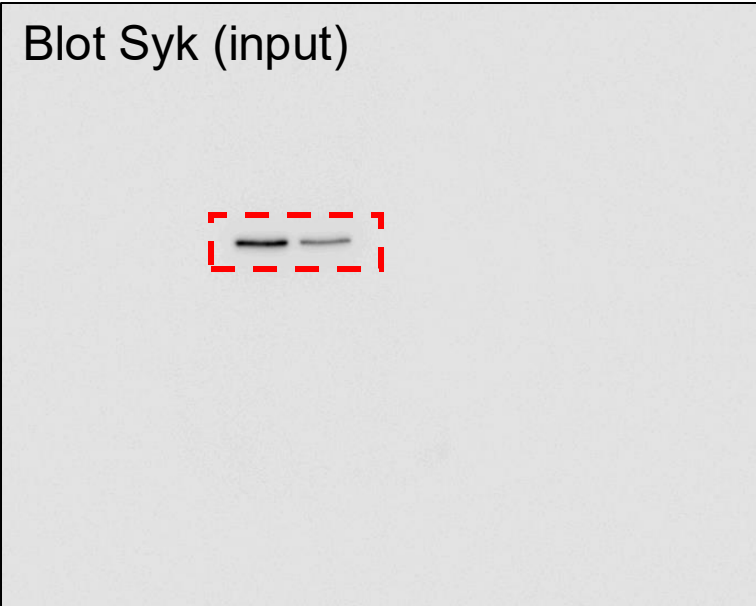

Supplement: Supplementary file 8 [file LSA-2025-03500_SdataF4.4.pdf]

**Figure 5**  
Panel A

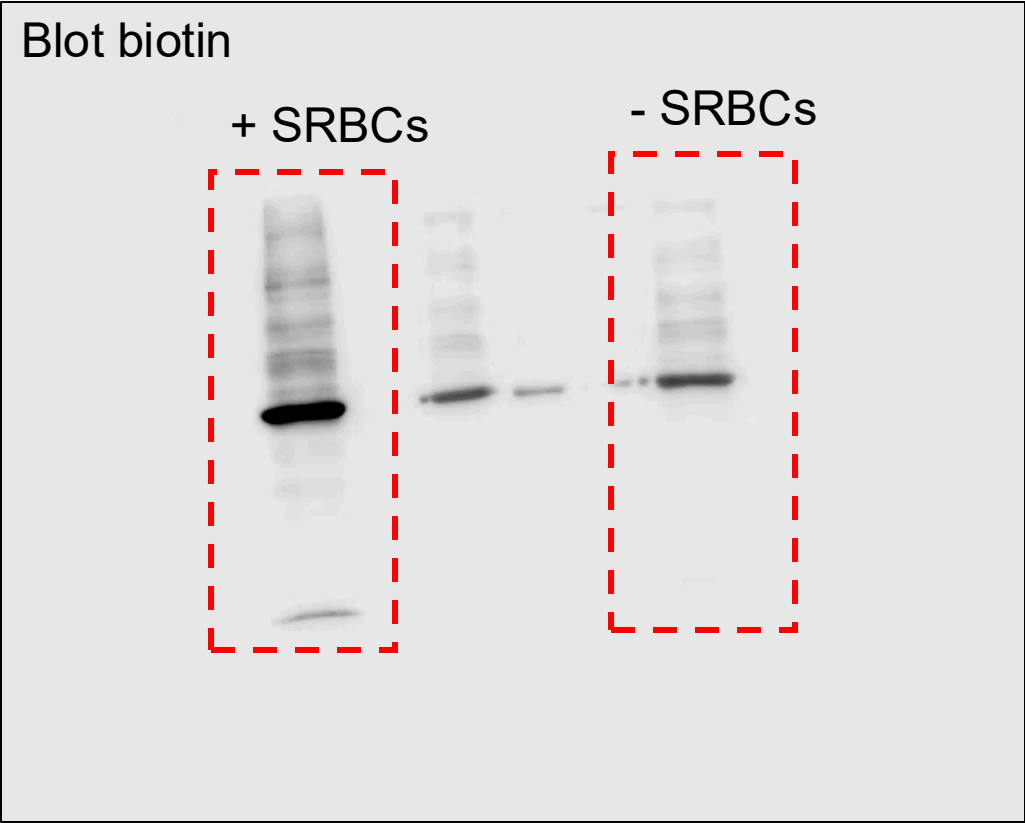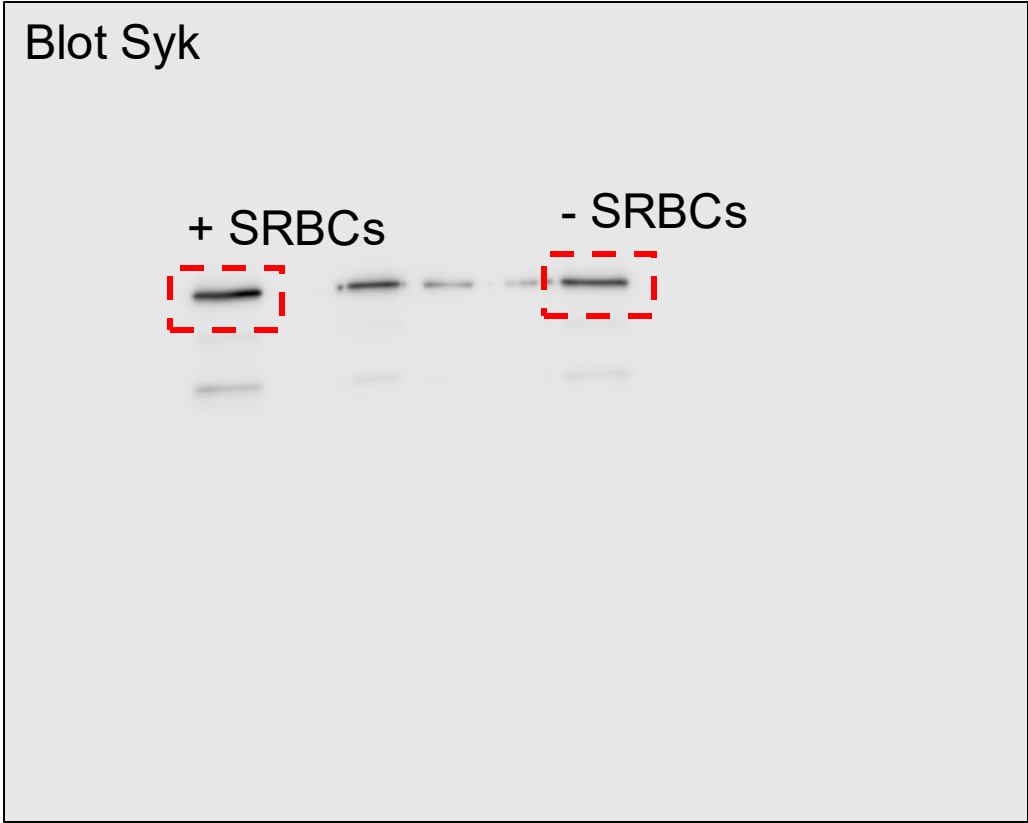

**Figure 5**  
Panel B

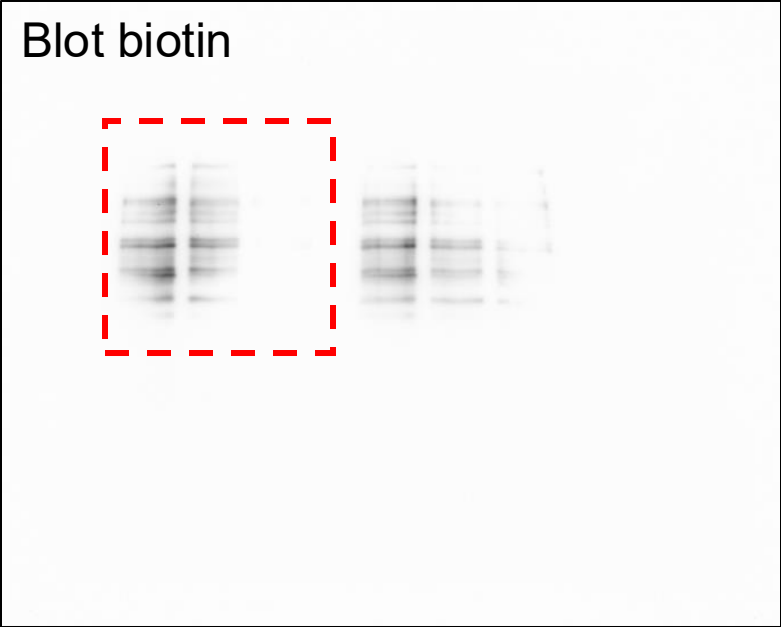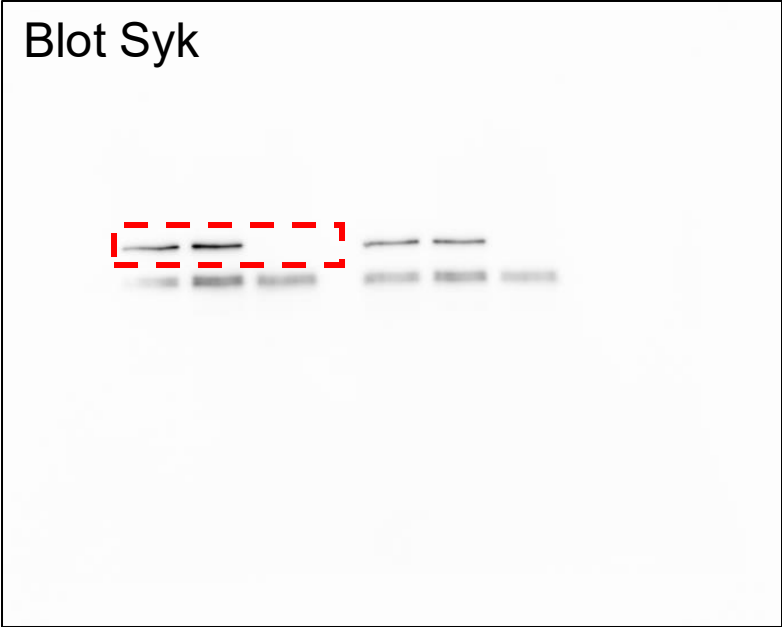

**Figure 5**  
Panel C

Blot biotin

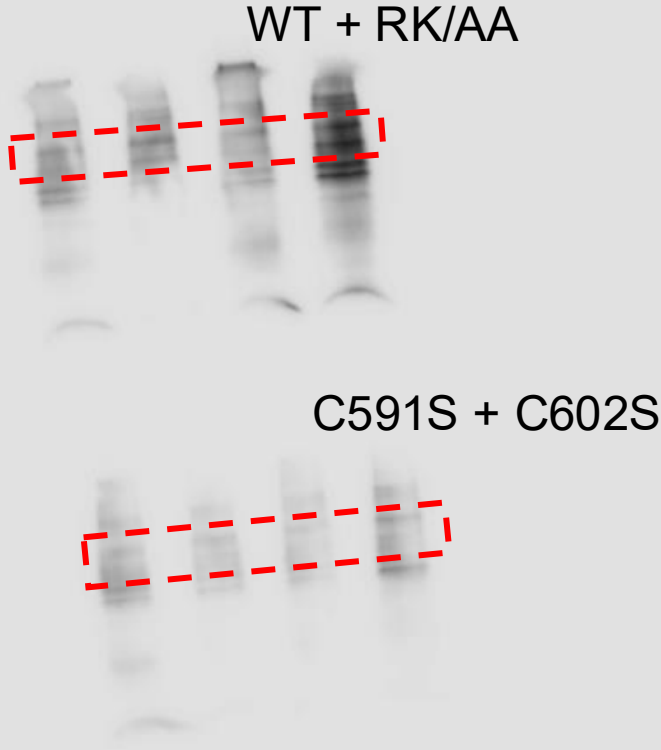

Blot pSyk

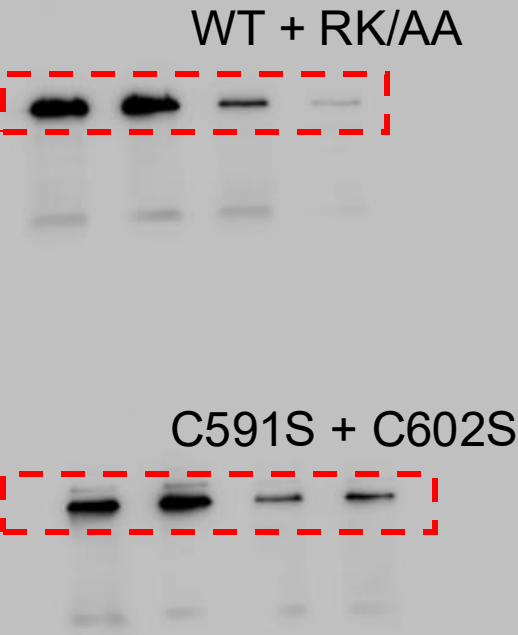

Supplement: Supplementary file 9 [file LSA-2025-03500_SdataF5.4.pdf]

**Figure 8**

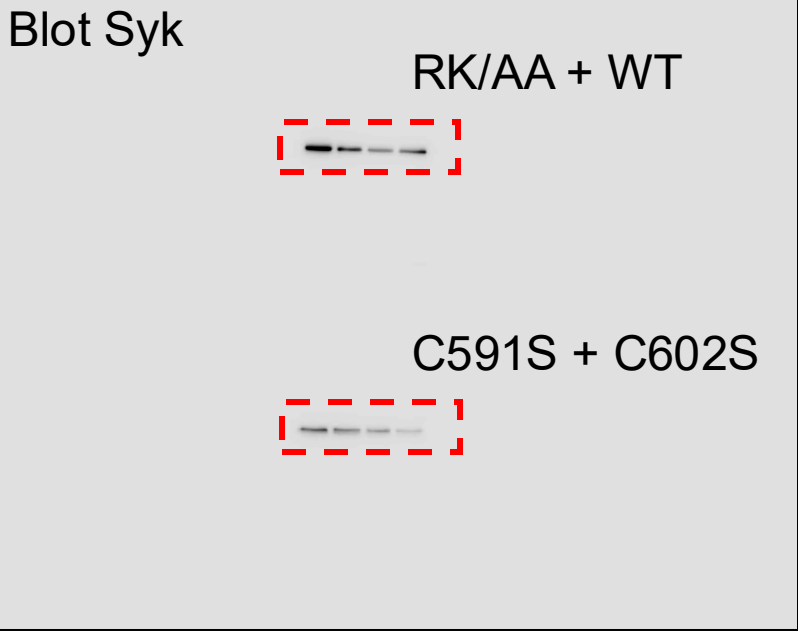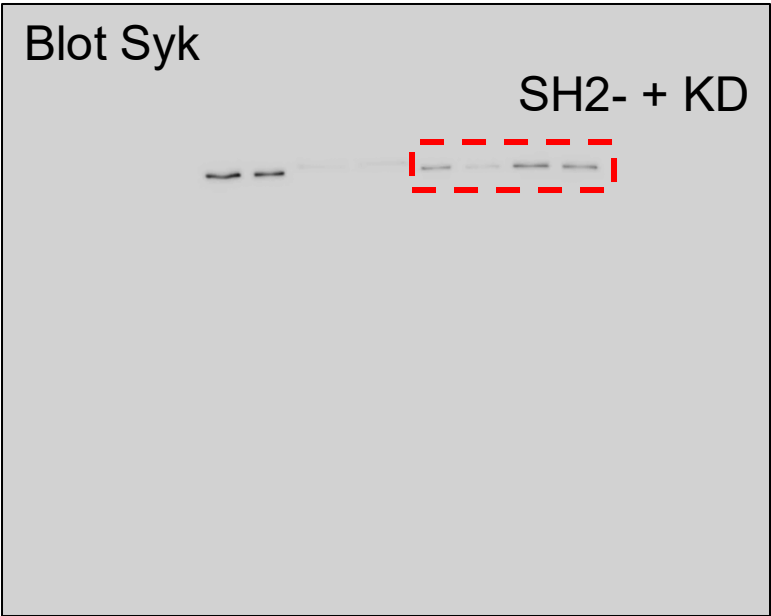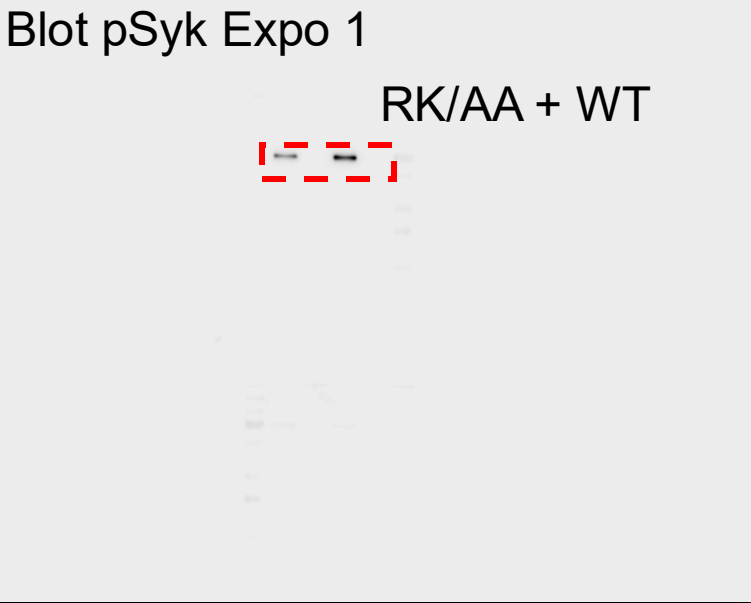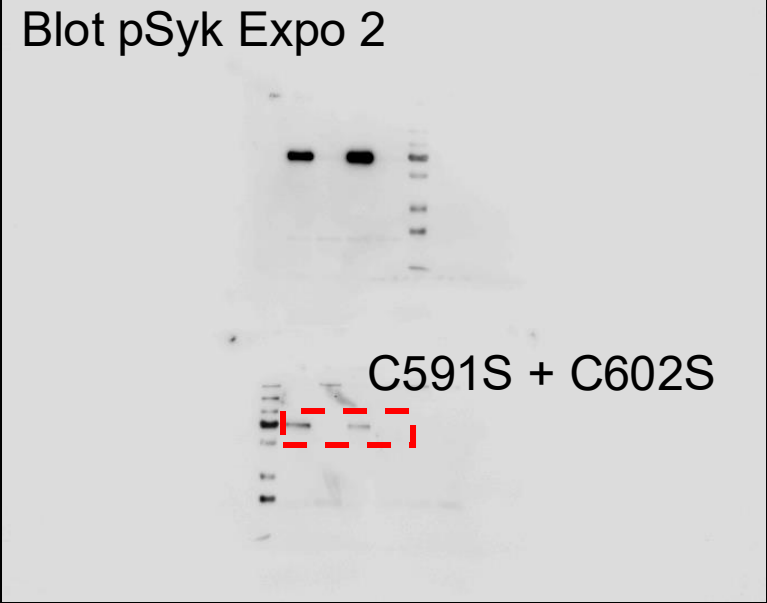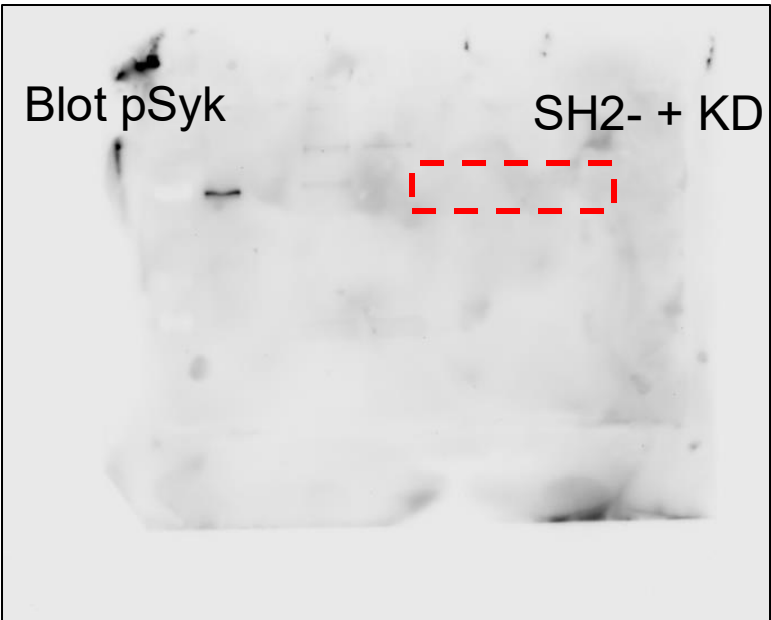

Supplement: Supplementary file 12 [file LSA-2025-03500_SdataF8.4.pdf]
