# Supplementary material for: Syk activation during FcγR-mediated phagocytosis involves Syk palmitoylation and desulfenylation
Source: Life Sci Alliance. 2026 Feb 4;9(4):e202503500. doi: 10.26508/lsa.202503500 (PMC12872395; doi:10.26508/lsa.202503500)
Supplement: Supplementary file 4 [file LSA-2025-03500_SdataF1.4_F2.3_F3.3_F4.3_F5.3_F8.3.pdf]

Figure 1

Panel A

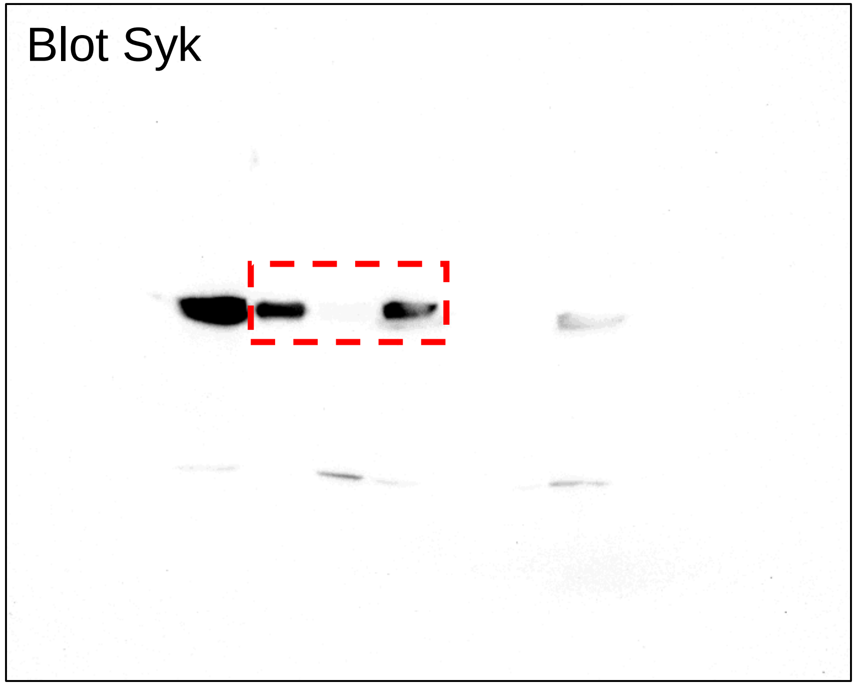

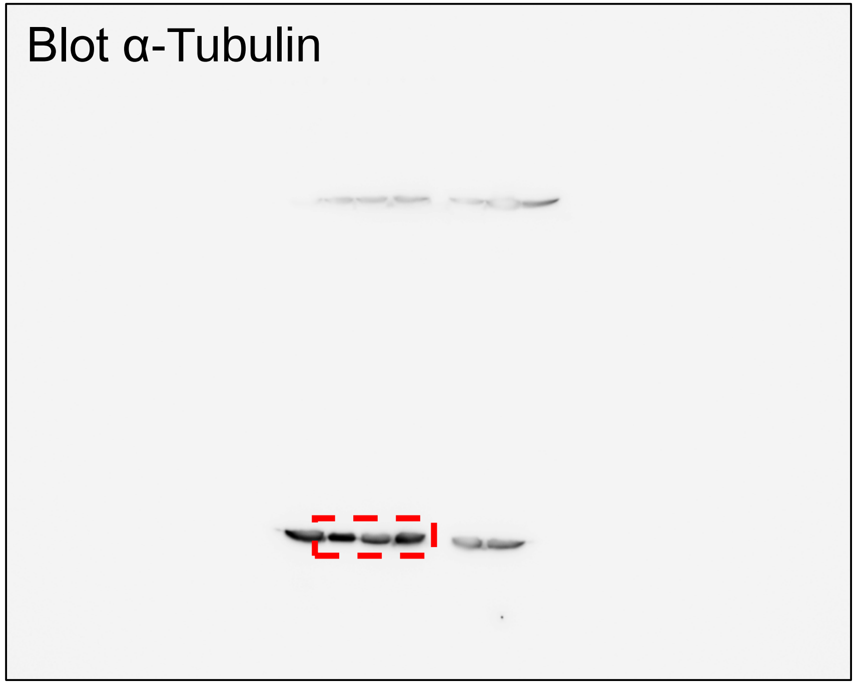

Figure 2

Panel A

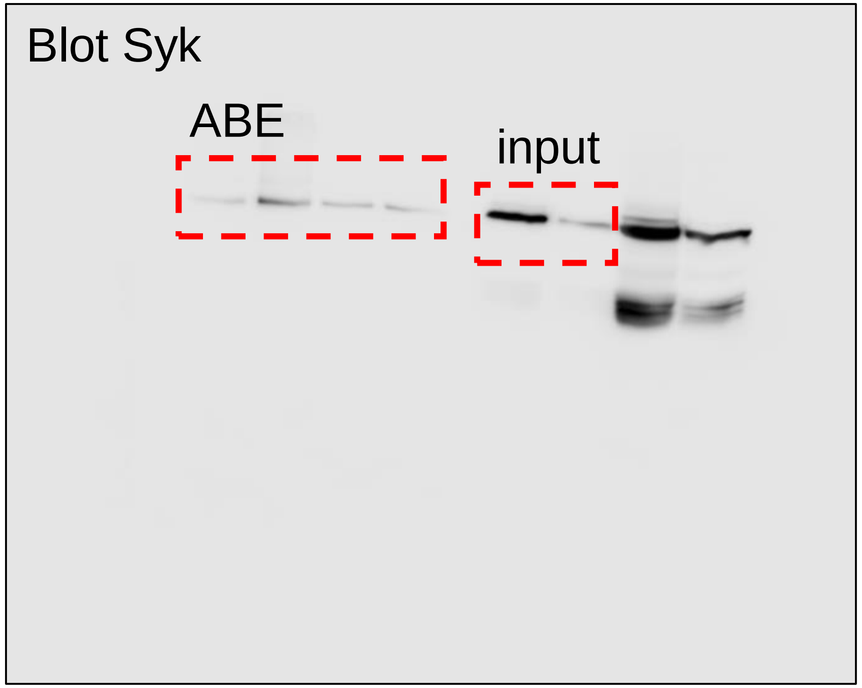

Figure 2

Panel B

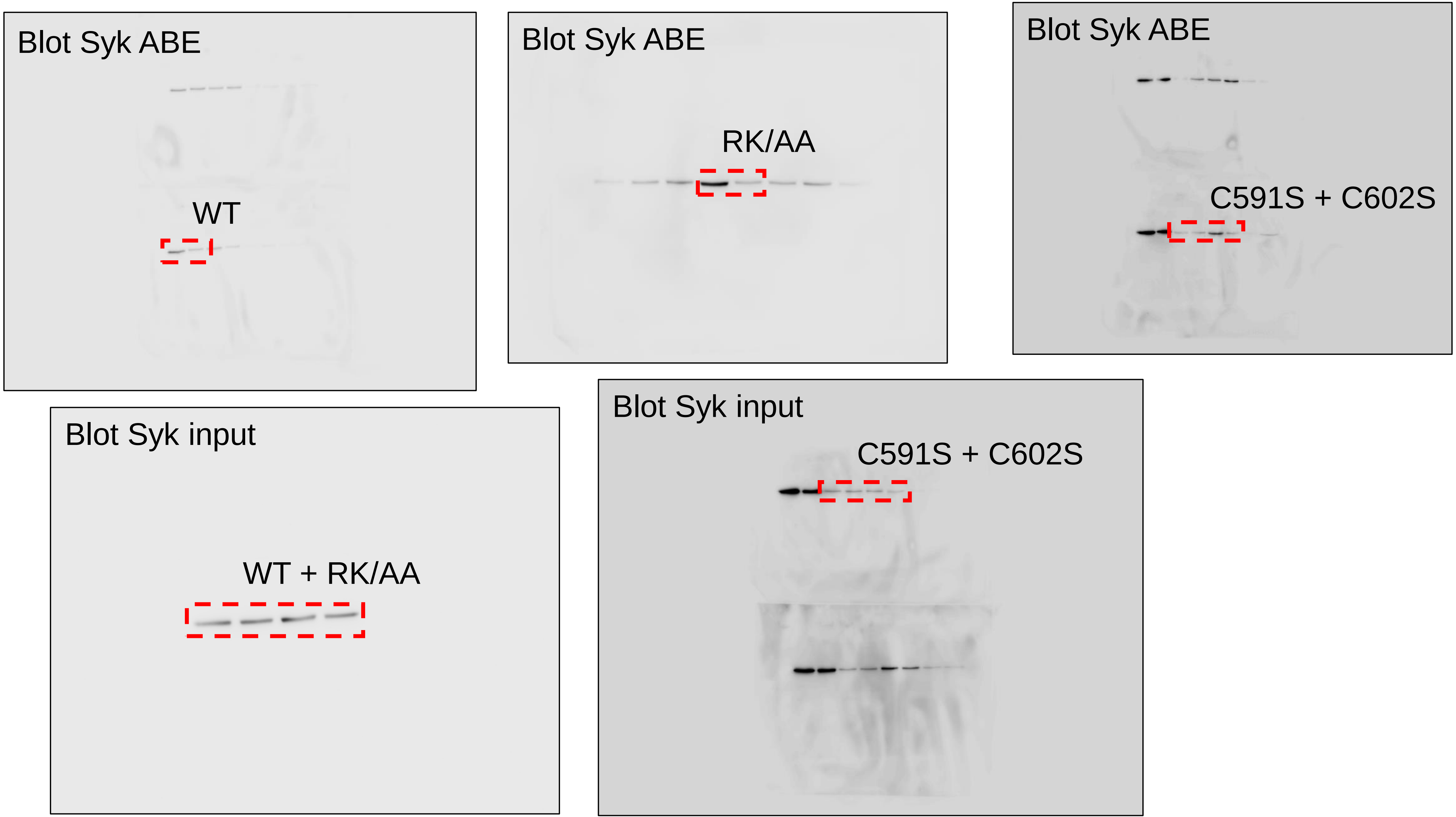

Figure 3

Panel A

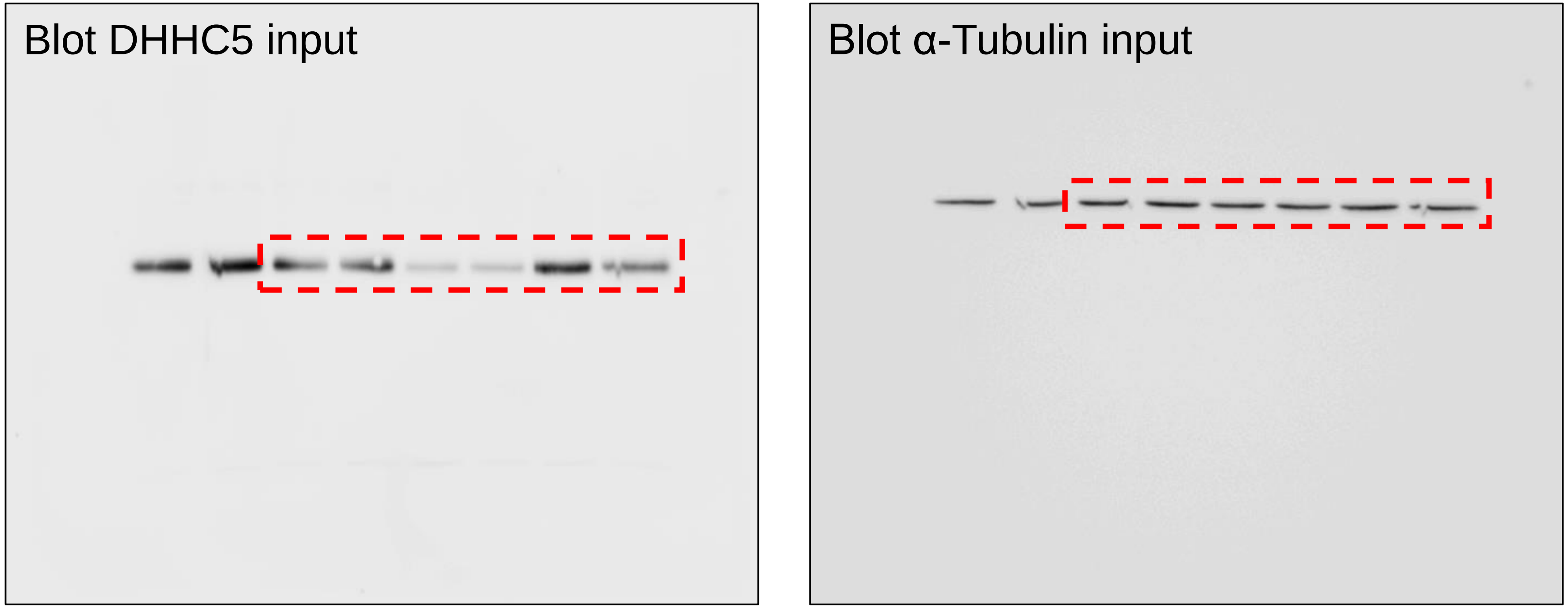

Figure 3

Panel C

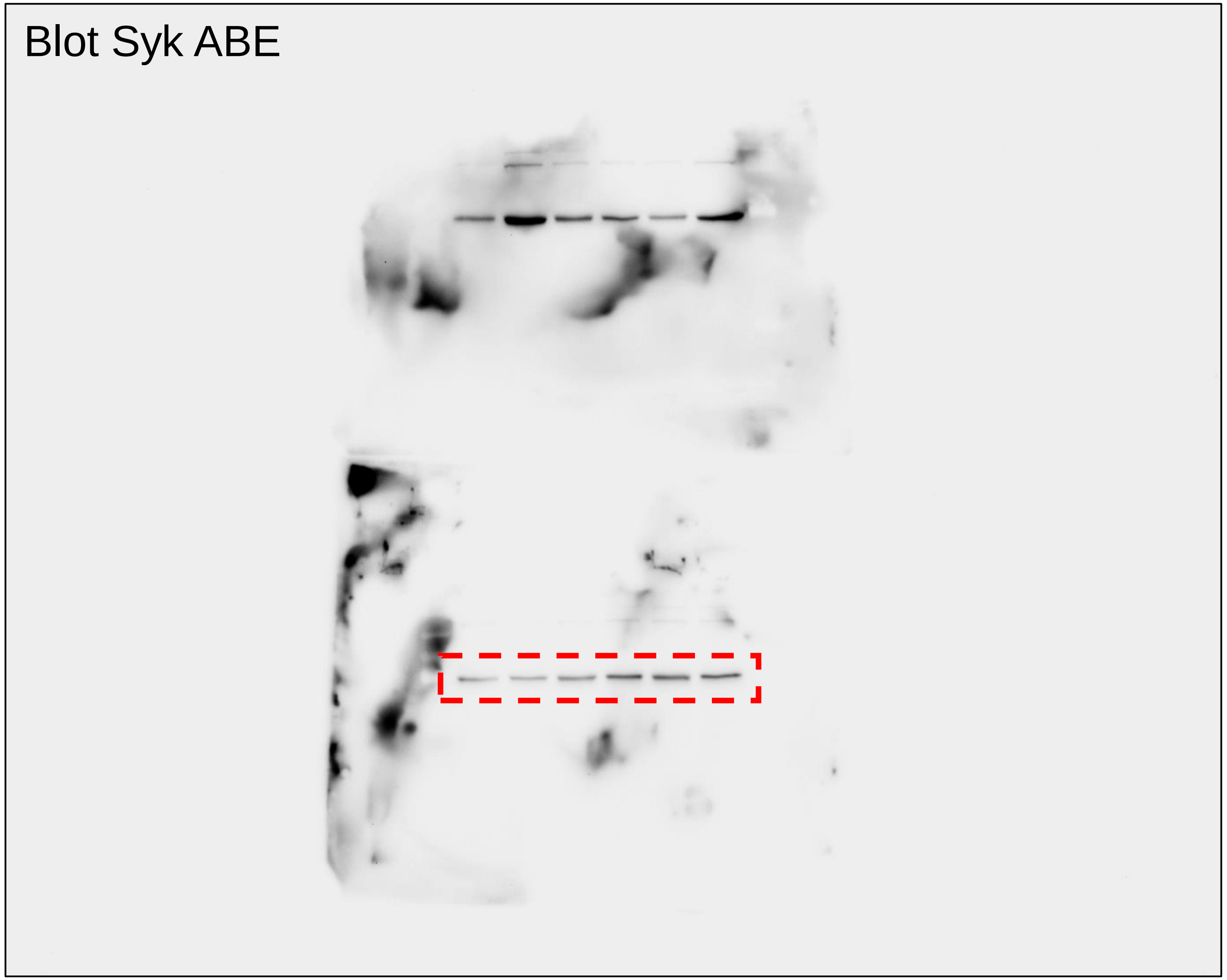

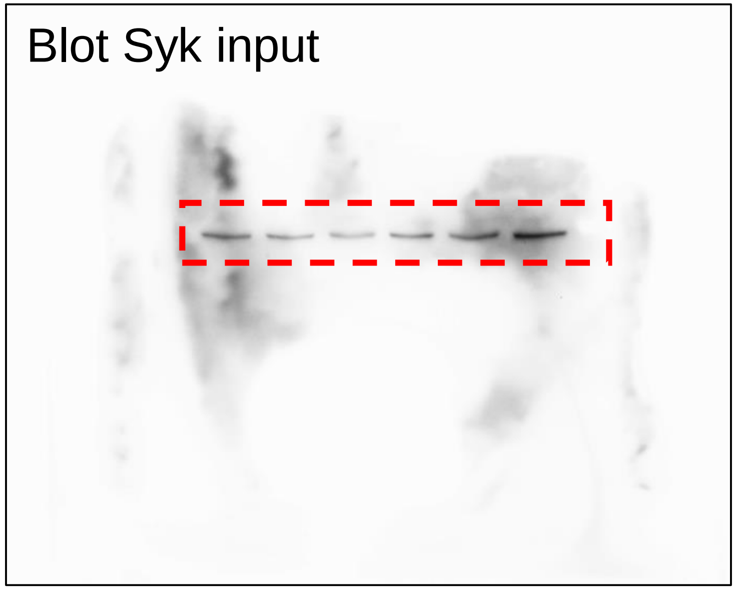

Figure 3

Panel E

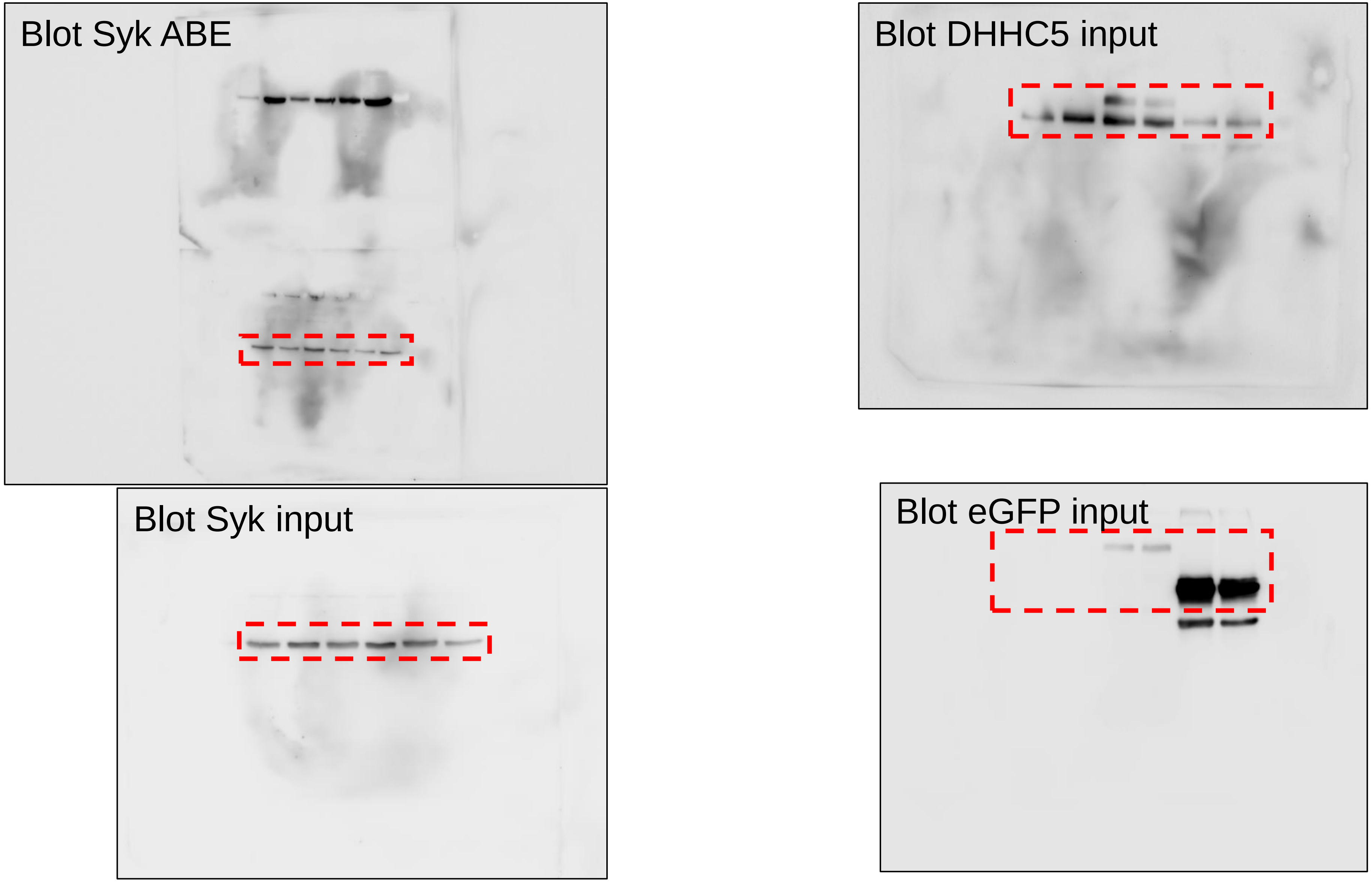

Figure 4

Panel A

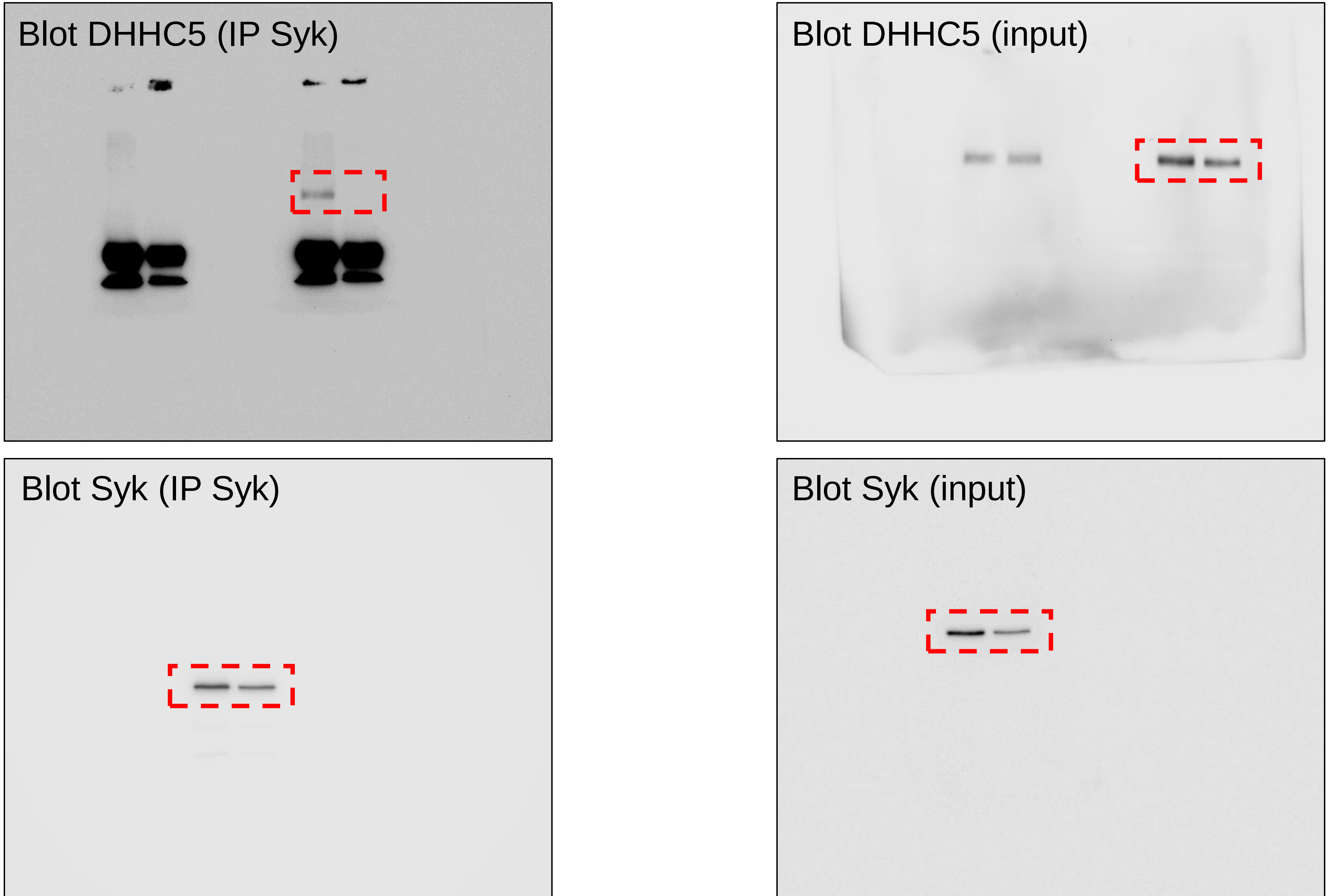

Figure 5

Panel A

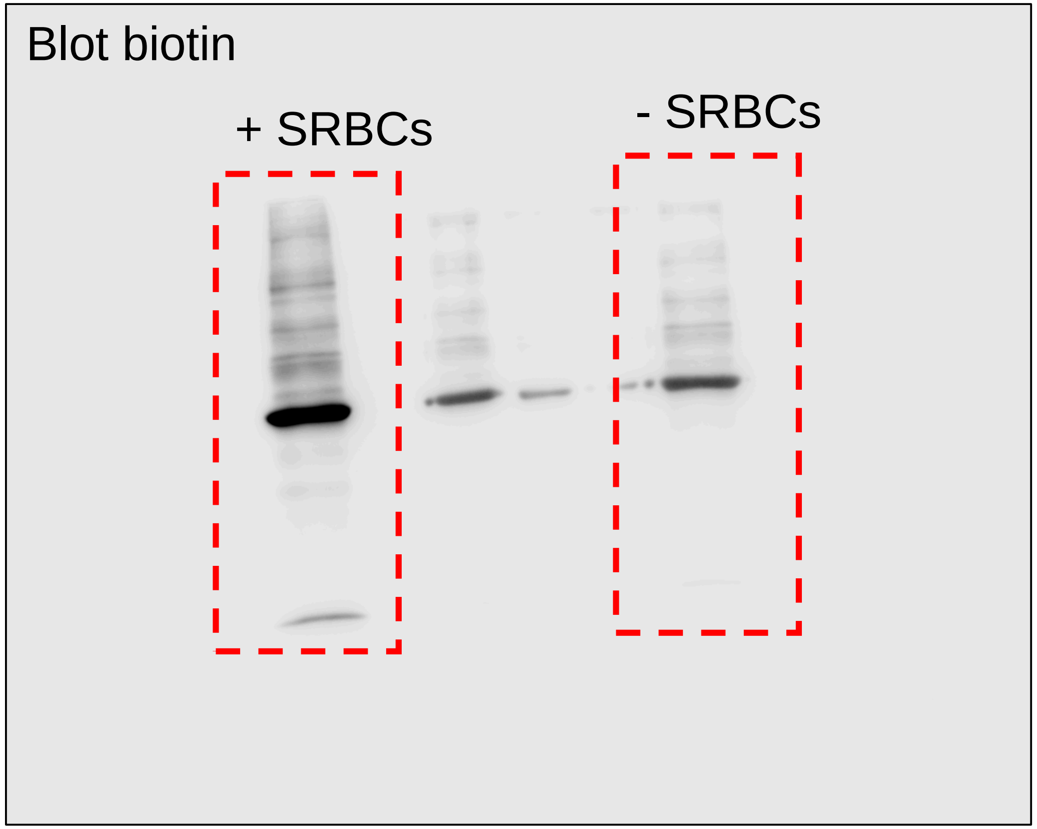

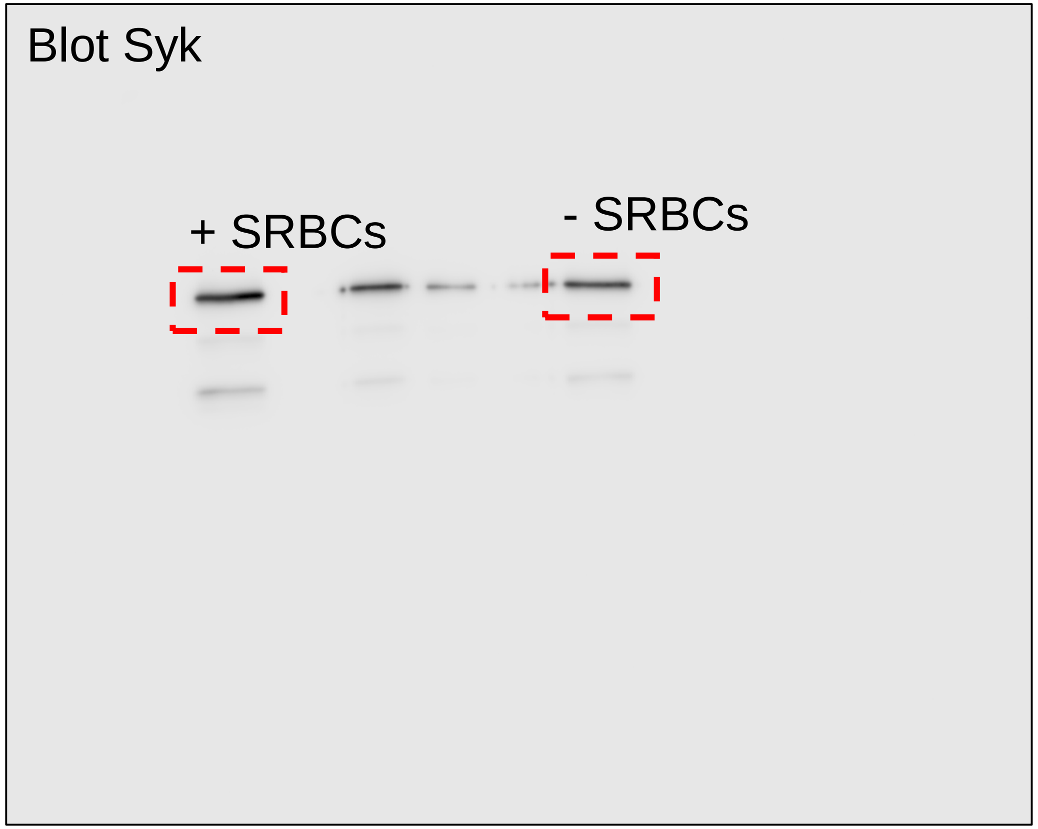

Figure 5

Panel B

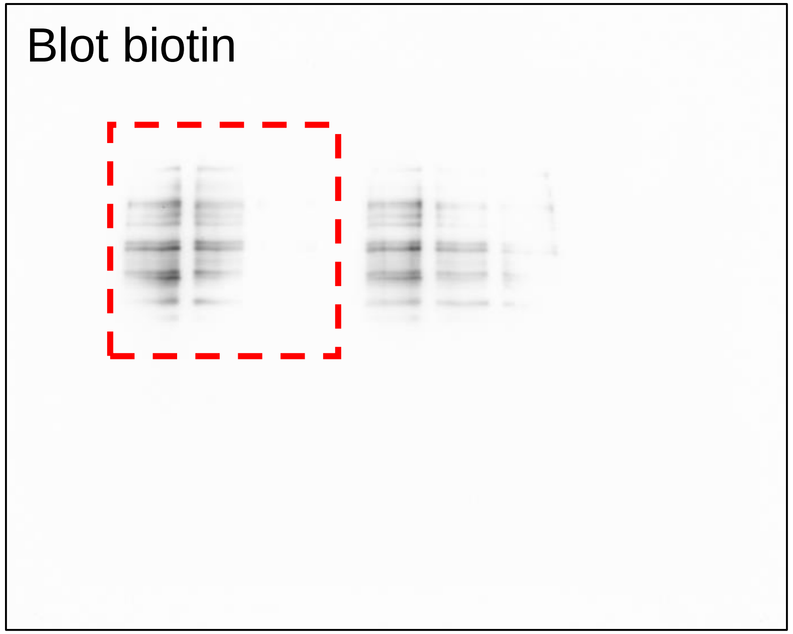

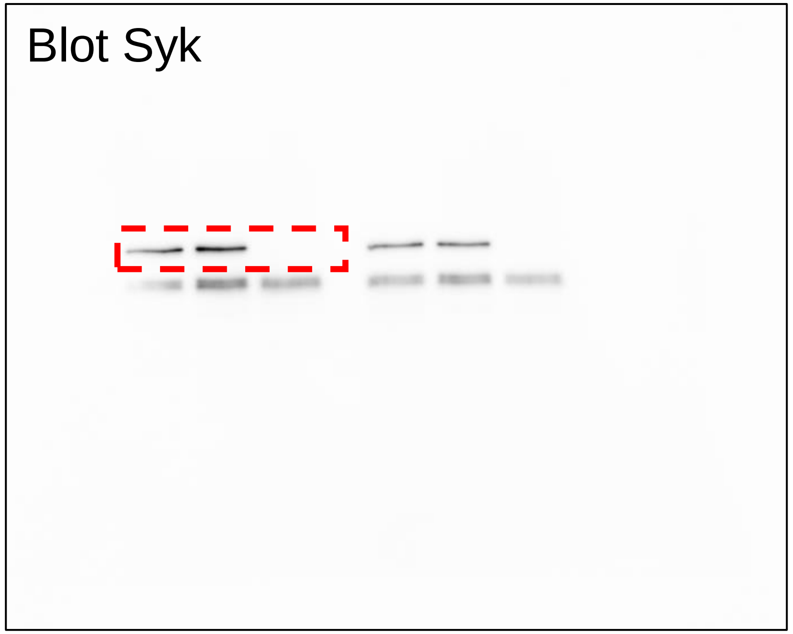

Figure 5

Panel C

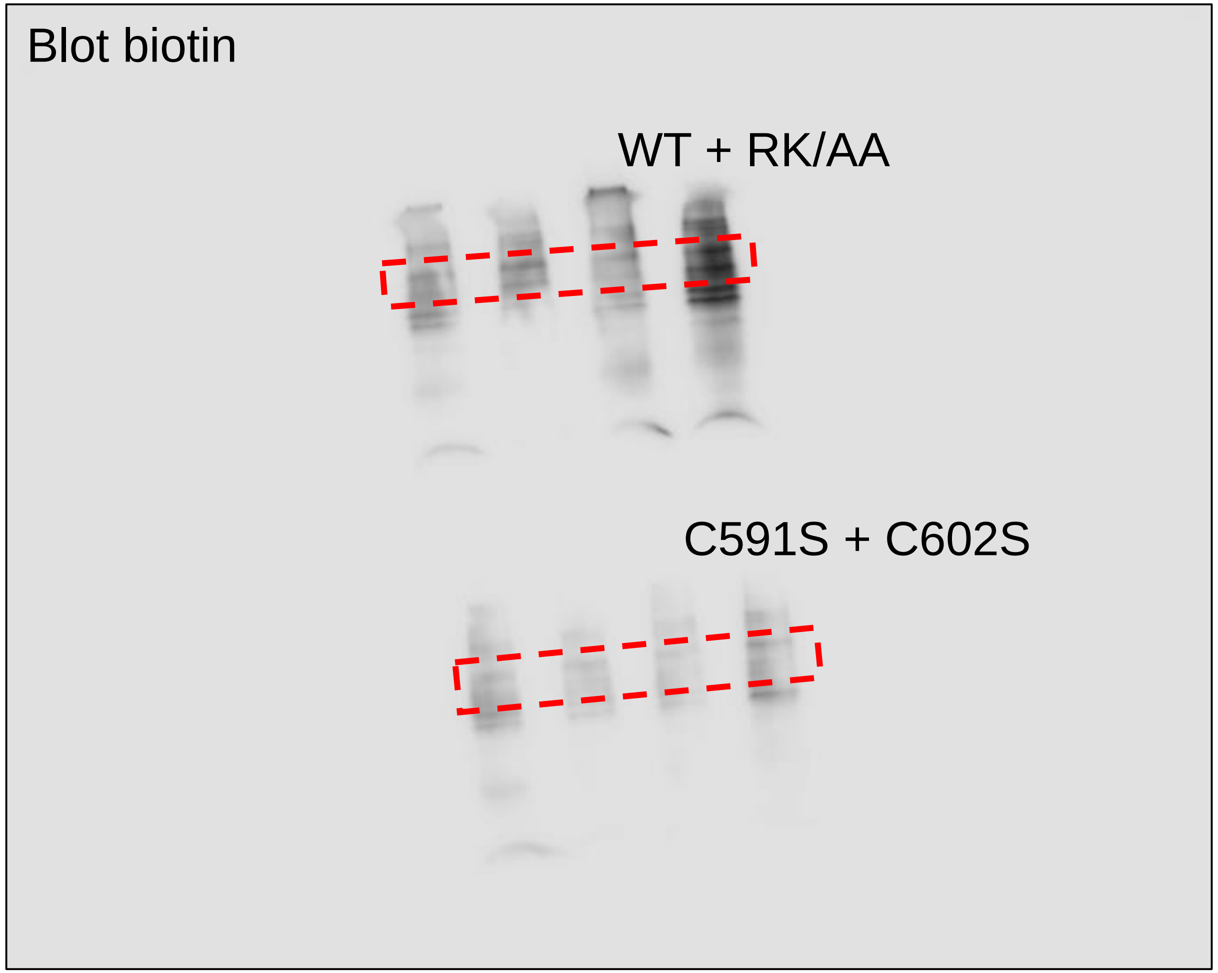

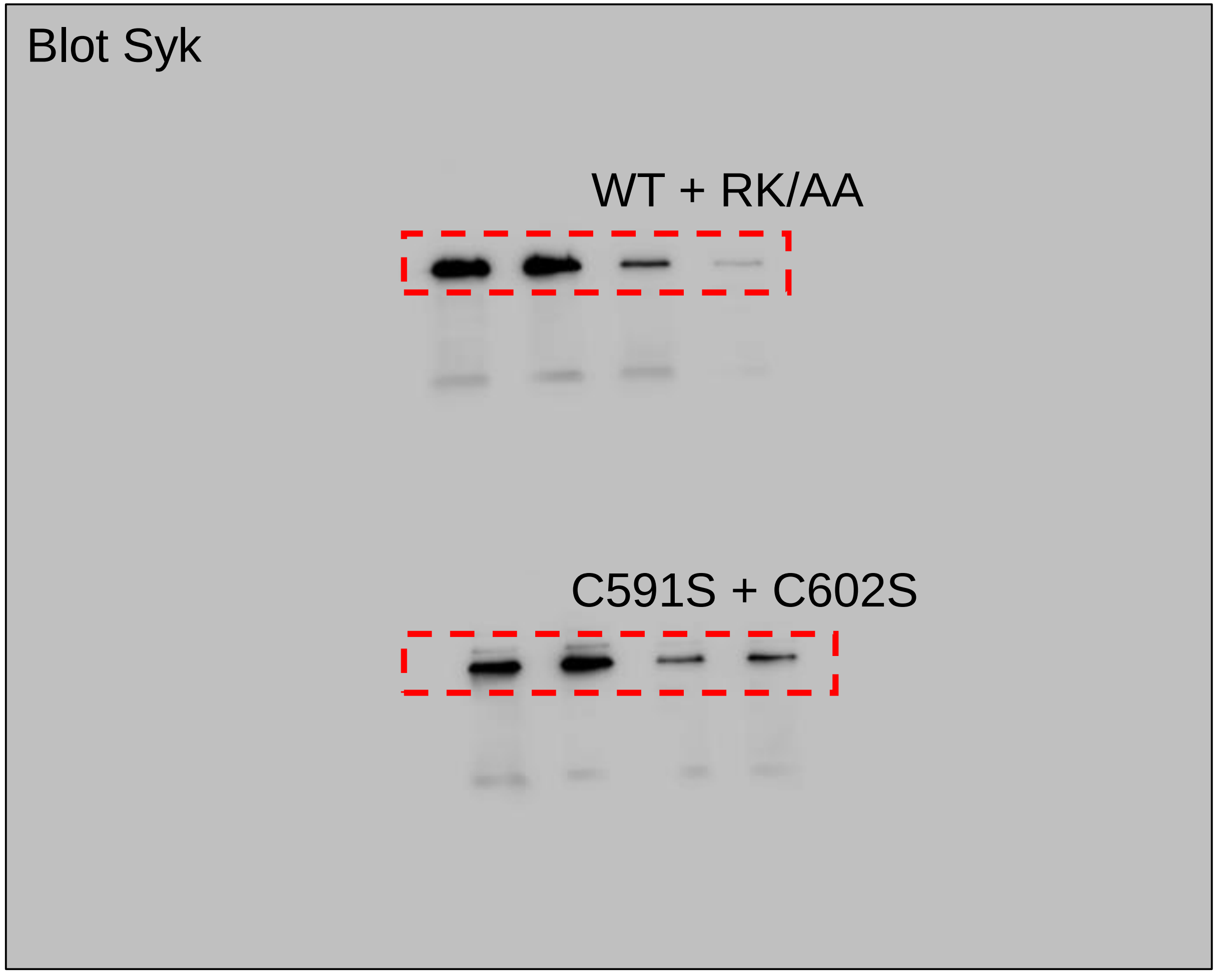

Figure 8

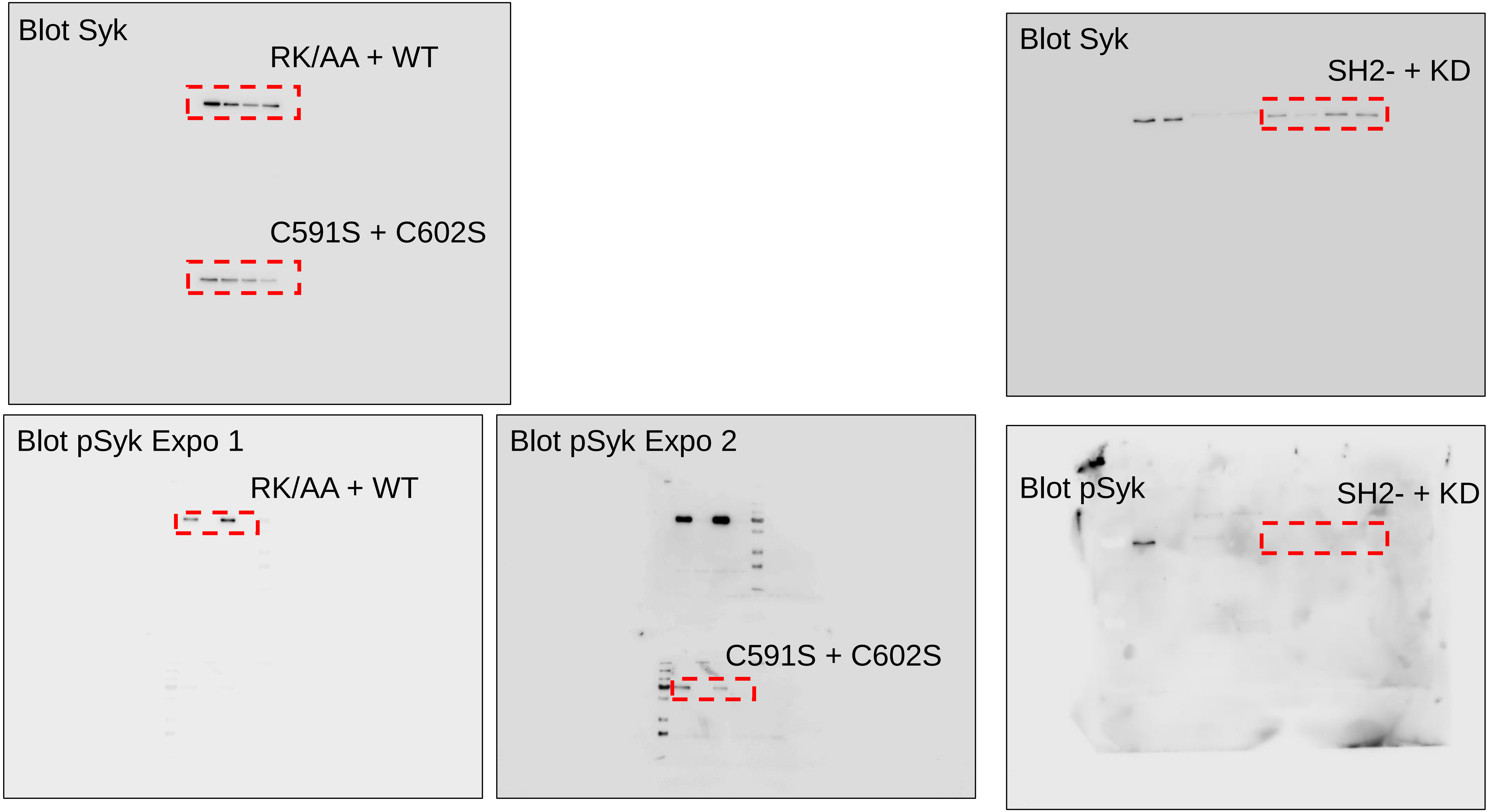
Blot Syk
RK/AA + WT
C591S + C602S
Blot Syk
SH2- + KD
Blot pSyk Expo 1
RK/AA + WT
Blot pSyk Expo 2
C591S + C602S
Blot pSyk
SH2- + KD
